# Medical Society of the County of Erie

**Published:** 1891-03

**Authors:** William H. Thornton

**Affiliations:** Secretary


### Society Proceedings.

### MEDICAL SOCIETY OF THE COUNTY OF ERIE.

Reported by WILLIAM H. THORNTON, M. D., Secretary.

The regular annual meeting of the Society was held at the rooms of the Young Men's Christian Association, Tuesday, January 13, 1891, at 10.30 o'clock A. M.

The meeting was called to order by the President, Dr. George W. McPherson, of Lancaster.

The minutes of the last semi-annual meeting were read and approved.

The report of the Committee on Membership was presented by the chairman, Dr. A. A. Hubbell. The following applicants were recommended for membership: Drs. L. B. Dorr, B. S. Bourne, Henry J. Mulford, E. A. Forsyth, H. Y. Grant, C. O. Chester, C. T. Wolsey, William A. P. Andrews. Several other applicants were not recommended, because they failed to comply with the by-laws and rules governing admission.

Upon motion of Dr. Callanan it was voted that the report of the Committee on Membership be received and the recommendations adopted.

The following applications were received and referred to the Committee on Membership: Drs. R. L. Patteson, W. J. Riehl, Earl G. Danser, William Dowlman, William P. Clothier, John J. McCullough, John Honsberger, E. H. Young, W. H. Chase, C. R. Jennings, J. F. Sell, M. J. O'Connell, James P. Wilson, W. H. Woodbury, and HughS. Townsend.

The newly-elected members present were then introduced to the Society.

The President appointed the following Committee on Nominations: Drs. A. A. Hubbell, E. H. Long, W. W. Potter, Joseph Fowler, and Edward Clark.

Drs. Crego, Long and Bartlett presented their resignations as delegates to the State Medical Society.

Upon motion of Dr. Callanan the resignations were accepted.

President McPherson delivered the annual address to the Society.

### THE PRESIDENT'S ANNUAL ADDRESS.

By G. W. McPHERSON, M. D., of Lancaster, N. Y.

*Gentlemen* — Members of Erie County Medical Society:

Being about to retire from office, it is expected that I should address myself to you in a more formal manner.

In the first place let me thank you for the uniform courtesy and kindness which you have shown me, and for the material assistance you have afforded me while presiding over your deliberations.

Instead of taking a definite medical topic and treating it analytically and historically, I have concluded that a more general survey of the field of your daily activities would be productive of more good.

Solon, one of the sages of ancient Greece, on being interrogated as to what constituted the ideal state or government, replied: "That in which an injury done to the least of its citizens is an injury done to all." What is true in high political and governmental organizations is true also in the best subordinate societies, so that, as far as it is known, an injury done to the least member of the medical profession is an injury done to all. If this be true, we can draw the natural inference that the promotion or advancement of one of the members is a good done to all. Out of this thought arises the idea of an individual *personal obligation* to the profession.

"It is the glory of our profession," says one writer, "that it is exclusively and rigidly scientific." It is built upon the works and laws, not of man, but of God — laws, too, not indeterminate, but capable of being classified and measurably controlled by the human mind. Scientific knowledge is imperishable and cumulative; therefore, each generation of doctors should be superior to their predecessors.

One way to advance the profession is to place a high ideal before the mind of the student prior to his taking up the study of medicine. Let him be a graduate of a college, a bachelor or master of arts, before entering upon the study of medicine. Do not rest satisfied until this is accomplished. The medical colleges are doing much to bring this into favor. They have within a few years elevated the standard of medical education, perhaps as rapidly as it is expedient, but they should not stop short of making the literary college the portal to the medical college. Let this be the rule, and the exceptions few, and every one who thus enters will, other things being equal, so comport himself that his life-work will be of a superior character. A good preliminary education is a powerful element in the professional struggle, for the physician is expected to have a large acquaintance with general scientific subjects and literature.

From the earliest times men were engaged in the healing art, and in precise proportion to the general culture of the people has our profession consisted of well-developed and superior men. Therefore, a physician should be a thoroughly educated man. If any class in the community should be well-trained scholars, familiar in particular with mental, moral and natural science, it is the physician.

The student need not expect to obtain all this training after beginning the practice of medicine. Even if he had the inclination to prosecute the studies, it would divert his mind from the cases he is called upon to treat, while the patient demands the *best he can do.* The art of treating disease with success will be his most useful study after graduating.

As you go out on your daily rounds, as much as it is possible, bring to the bedside a clear mind and good judgment, and an ever-increasing valuable experience, for it is experience and skill that the public seek.

In the next place let me remark that every physician in the State should belong to a medical society, even if the law had not made it obligatory, for beside the individual benefits which accrue to the member, a genuine professional spirit is generated that constantly minimizes all that is unprofessional. A man is unworthy to be a member of any society who is not willing to sacrifice something of his personal right for the good of the whole. Acting on this line, it has always been considered the proper thing to do in the regular profession, when a new remedy or treatment is found out, to publish it to the world, so that all physicians can give humanity the benefit.

Thus, by advanced medical knowledge and skill, by better ventilation and sewerage, and the isolation of patients, the average length of human life has been increased. What has been added to each life in the last two hundred years is equal to the saving of a million of lives. This applies only to the civilized nations. This work will still be carried on, and you must do your share of it. The physician's life is a busy one, and there is but little room for the idler. He would soon find the place occupied by some who loved to work, for the work *must be done.*

This diversity of interest among physicians, on account of family relationships, and the multitudinous bonds of society calling two physicians in the same family, leads me to emphasize the necessity of great care in your business relations with one another. Observe both the rules and the spirit of the code of medical ethics. It is intended to be for a saving of time in the ever-changing relations of society, but in no sense is it subversive of the golden rule. The code only shows the application of the golden rule to individual cases, and is founded on equal rights and privileges. Show that you belong to a noble profession, even under provocation. Honor and duty require you to do right, not for policy, but because it is *right.* Courtesy, truth and justice should mark every step of your career.

"You must be charitable, not censorious, self-effacing, not self-seeking, and you must try at once to think and to do the best for your rivals and antagonists that can be done." As Lincoln said concerning things political, "With malice toward none, with charity for all, with firmness in the right, as God gives us to see the right, let us strive on to finish the work we are in."

The great glory of our profession is the practical use which has been made of knowledge by its members. The very soul of its life seems to be a constant inquiry: How can suffering be alleviated? How can disease be removed? How can life be made more comfortable and prolonged? To accomplish this, no investigation can be too minute, no labor too severe, no exposure too dangerous.

The true medical profession discards all *schools*, or *cliques*, or *parties*. It is as old as civilization. It never forgets anything. It neither receives nor discards anything because it is old, or because it is new, but following the precept, "It proves all things and holds fast that which is good."

Or in the language of Professor H. C. Wood, "There are but two bodies in the field of medicine, physicians and the *paths*. Try to make the public understand this. If you must have some name," said he, "let it be that of a common-sense doctor, who, brought face to face with the problems of life and death, is willing to save life in any way, who acknowledges no boundaries, no sects, no schools, but who searches heaven and earth to find means to relieve suffering and cure disease."

Some of you do not half appreciate the fact that you are in the line of the regular work of the profession where all methods and remedies, which *true science* may bring forward, can be used without a change of principle or a sacrifice of your manhood.

Who would think of having anything but a regular work on anatomy, physiology or chemistry?

You may call the chemicals by other names, as is often done by designing men, in order to appear different from the great majority. You may use different names for the muscles, bones and nerves than those used in our standard works on anatomy. The same may be said of physiology, materia medica and surgery. But you should not desire, for selfish and *personal ends*, to depart from the great beacon lights of true science.

"What is the best treatment known to the world for a case like the one before me? If you give him that without regard to creed or boundary, that is practising rational medicine."

Bear in mind that competent medical men all over the world, in the interest of truth and suffering humanity, investigate and test all so-called systems when they arise, and the conjoined result gives us a true, common-sense verdict. This is being done now with the treatment for consumption proposed by Dr. Koch, of Berlin. The verdict will be given when thoroughly tried. In the meantime hold your souls in patience.

Let me direct your attention to some of the elements of success that as you advance to greater and still greater degrees of success as individual practitioners, we all, and the profession at large, may be benefited by your advancement and promotion.

Sophocles said, "Fortune never helpst he man whose courage fails." And I read somewhere a concise statement of the reasons why, sometimes, a good doctor does not reach success: "It sometimes arises from the superabundance of qualities in themselves good; as, from a conscience too sensitive, a taste too fastidious, a self-forgetfulness too romantic, and a modesty too retiring." The opposite extremes would make a man a disgrace to any profession. Entertain loftier sentiments and make nobler efforts to build your fame on a strong foundation. Do nothing that will not stand every test.

Chauncey M. Depew, when speaking at the commencement exercises of Syracuse University last Spring, before the graduates in medicine, used language which I think is worthy to be repeated:

> "Every conscientious doctor presents a picture full of romance in the realities of his experiences, of character which strengthens the weak, of breezy hopefulness which encourages the despairing, and of deeds and thoughts which so sanctify him that his presence elevates society."

"The destiny of man is largely in his own hands," said Dr. J. W. Hamilton, "and a man's energies are set in just equation with his *opportunities* and *worth.* Success comes to the man of indomitable perseverance and worth. There is no road that will avoid labor, and the measure of a man's success is the measure of the motive and power by which he is moved." And Dr. Clark, one of England's foremost physicians, said, "the man to succeed must believe that *labor is life*, and successful labor is *life* and *gladness*, and that success with high aims and just objects will bring to him the fullest and happiest life that can be lived upon earth."

Some of the members of this Society whose demise we deplore, had a national reputation, I might say a world-wide fame; others have gone to other fields of usefulness. Among the *many*, allow me to mention the names of Flint, Hamilton, Miner, White and Rochester. Here is an illustration of how the elevation of the few can result in good to the many. It is no flattery when I say that there are many of the present members of the Erie County Medical Society, who have already attained to great distinction. This is a foreshadowing of still greater professional triumphs. Then, let each member seek to *improve himself*, to succeed according to the possibilities that are in him, till it shall be seen that there is a general advance all along the line. As the field and forest, by leaf and flower, show the beneficent effects of the rain, and the light and warmth of God's sunshine, so may we as members of a lofty profession show that we are living, growing, rising and flourishing to meet the ever-increasing demands of this great city and the age.

Upon motion of Dr. STORCK it was voted that the thanks of the Society be tendered to the President for his able address, and that a copy be requested for publication.

The Treasurer read his annual report, as follows: Receipts, $583.48; expenditures, $222.00; balance in treasury, $361.48.

Upon motion of Dr. CALLANAN, it was voted that the report be received and referred to an Auditing Committee.

Dr. ABBOTT, chairman, presented the report of the Committee on List of Members, and distributed proof lists of the names as found by the committee.

Upon motion of the committee it was voted that the printed list distributed, together with the names of those today elected members of the Society, be the official list of members of the Medical Society of Erie County; also that the names of the ex-presidents be printed in a different style of type from the other names.

Upon motion of Dr. JOHN CRONYN, it was voted that the report of the Revising Committee be received and adopted.

The Auditing Committee reported that they found all the accounts of the Treasurer correct.

Upon motion of Dr. POTTER, it was voted that the report of the auditing committee be adopted.

Dr. E. STORCK, chairman, presented the following

### REPORT OF THE BOARD OF CENSORS.

*To the Medical Society of the County of Erie:*

Since the last annual meeting of the Society, a State Board of Medical Examiners has been created, by a law passed by the Legislature of this State, and will go into operation September 1, 1891. Graduates of medical colleges of this and other states, and those from foreign countries, will have to submit their diplomas to that board, be examined and acquire a license of the Regents of the State University, before they are allowed to practise medicine or surgery within this State.

Diplomas endorsed by the faculty of any medical college of this State, after June 1, 1890, are invalid, and the holder thereof is not legally authorized to practise in this State.

The board of censors was directed by the Society to bring an action against some practitioner who has failed to become a member of one of the county societies after having been notified to do so, in order to test the legality of the law of 1828, which, as it is claimed, has not been repealed by any medical law since passed, namely, to make it compulsory for any physician to become a member of a legal medical society of the county where he practises. We have deferred such action until the question has been submitted and decided by the State Medical Examiners and the Regents.

The section of the medical law, giving medical societies the right to discipline their own members, is still in force and should be more rigidly enforced. Your board has information that among the members of this Society, there are some who persistently violate its by-laws and the code of medical ethics, by being connected in so-called medical institutes "or companies" that secure by quackish advertisements and pamphlets some business in and outside of the county. If sufficient proof is furnished of their offense, the board will prefer charges against them to the Society at its next meeting.

The "advertising doctor" has, in fact, become quite an institution in this city and state. The newspapers are full of "ads." of "specialists" and "medical institutes," that promise to cure all ailments of mankind by "new and approved methods." It seems as if the whole doctor business will soon be done in this way. The idea has already become prevalent among the younger class of physicians, that in order to be successful in the profession and make money fast, one has to seize a specialty or step out of the regular profession, and advertise his skill and success either in the advertising or local columns of the newspapers. This mania threatens to become prevalent and contagious, several of our regular graduates have been affected, and I am sorry to say a member of the Board of Censors has lately also been seized by it. What are we going to do about it? The question has repeatedly been asked: Why do the censors not interfere with these advertising doctors? Simply because they can't.

These managers of medical institutes know very well how to evade the law, or rather how to manage their business in compliance with the law. They advertise that "a staff of European physicians," or a corps of consulting doctors from "Boston and New York," are attached to their medical institute; they publish marvelous cures that have been effected by them, but when we look up the Medical Registry, we never find the names of any such celebrities, but only the name of some young graduate of unknown fame; or of an old country physician who is legally authorized to practise under a license granted by some county society before we had any medical laws, and under the protection of one or both of them, these impostors of "medical institutes" are allowed to humbug the people and fleece the dummies out of their money. A short time ago the board served notice upon three specialists, whose names were attached to the "ad." of one of those "catarrhal institutes," but did not appear upon the Medical Registry, that they would be prosecuted for violating the medical laws of the State; a prompt answer was returned by their attorney, saying: "Don't trouble yourself with any prosecutions; this concern is sailing under the flag or diploma of a regular graduate, *a member of your Society*." Those signed to the advertisement were but managers. That member is more an object for commiseration than for discipline. The success of all such institutions simply depends upon extensive advertisments like that of *patent medicines*, and shows that anything can be made to pay if you only advertise it well. There are plenty of people that believe with the old settler, "it must be true, for it stands in the newspaper," and as long as the newspapers can divide the profits with those "institutes," they will not interfere with their business and expose these impostors.

Some of these *dyspeptic* and *catarrhal* institutes have lately adopted quite a business-like method to make money out of their patients; they advertise to treat them cheap by contract, say $5.00 a month, or less if a few months are taken and cash paid down; this is quite an inducement for some suffering with a chronic catarrh or some imaginary ailment. One of them, who is now in the last stage of tubercular laryngitis, said to me the other day: "I had treatment for several months; I had taken ninety-eight bottles of medicine, and it cost me only $25.00." In addition to that he had the satisfaction of having his name and his picture appear in the newspapers, and held up as a *well man*.

Probably no law can be secured to prevent the people from being humbugged "*multus vult decipe*," but it should be made a misdemeanor for any physician, legally authorized to practise, to advertise in a quackish manner in the public press, or by pamphlets, or associate himself in business relation with the managers of bogus medical institutes that are guilty of obtaining money under false pretenses.

The Regents should have power to revoke his license to practise. Our State Medical Societies should urge upon all county societies to report the names of such practitioners registered in their County Clerk's office where they are engaged in such irregular practice, to the faculty of their alma mater that has granted their diploma and that they disgrace. Their names should be stricken from the roll of its alumni, and if they are members of some legal societies in the State, they should be expelled from its membership.

Your board would further report that several persons practising in violation of the law in this county, have been notified by the Censors to desist from doing so, but as they still continue to practise, their names will be given to the District Attorney, who will bring the cases before the next Grand Jury.

There is another class of impostors that prey upon suffering humanity under false pretenses ; they go by the names of Christian Scientists or healers and clairvoyants. Their practice does not strictly come under the medical law, but rather under the Penal Code. Our efficient police authority will, before long, give them an opportunity to defend themselves on charges of a criminal offense. They are still more dangerous to the public than the "catarrhal institutes," as they fleece the people out of their money under the protection of the Christian religion or so-called science.

The Board of Censors.

Edward Storck, *Chairman.*

Upon motion of Dr. Hoyer, it was ordered that the report of the Board of Censors be received and published in the Buffalo Medical and Surgical Journal with the report of the meeting.

Upon motion of Dr. Phelps, it was voted that a printed circular be issued by the Society, and sent by the President to such practitioners in the county as were mentioned in the report of the Committee on List of Members, and who were not members of the Medical Society of Erie County.

Dr. W. W. Potter offered the following preamble and resolution, which was unanimously adopted:

WHEREAS, The by-laws of the Medical Society of the State of New York, require that each society entitled to representation therein, shall purchase each year a number of copies of the State Society Transactions, equal to five times the number of delegates it is entitled to send to the State Society, therefore

*Be it Resolved*, That the Treasurer of this Society is hereby instructed and required to disburse annually, a sufficient sum to comply with the aforementioned rule of the State Society, to be reckoned and accounted for as a part of the current expenses of this Society, payment to be made on the voucher of the Treasurer of the Medical Society of the State of New York. This order to take effect immediately, and to include the Transactions for 1890.

Dr. ROSWELL PARK extended an invitation to the physicians present to attend a demonstration of the use of the Koch lymph, at the Buffalo General Hospital, at three o'clock P. M.

The Committee on Nominations reported in favor of the following officers:

*President*, E. C. W. O'Brien; *Vice-president*, W. H. Gail, of East Aurora; *Secretary*, William H. Thornton; *Treasurer*, F. W. Abbott; *Librarian*, Lucien Howe.

*Censors*, Edward Storck, R. L. Banta, Henry Lapp, Clarence; M. B. Folwell, E. H. Ballou, Gardenville.

*Membership Committee*, A. A. Hubbell, F. H. Potter, John A. Pettit.

*Delegates to State Medical Society*, to fill vacancies, B. H. Grove B. H. Daggett, Edward Clark.

Dr. THORNTON stated that having served as secretary for five years, he was not a candidate, and requested that another name be substituted for his.

Upon motion of Dr. HUBBELL, Dr. E. H. Long was nominated for secretary.

Upon motion of Dr. DORLAND, it was voted that the Secretary cast the ballot of the Society for the officers, as presented by the Nominating Committee, as amended above.

The ballot was cast as directed, and the President declared these gentlemen duly elected.

Dr. ABBOTT moved the adoption of the resolution introduced by him at the last regular meeting of the Society, that Article II. of the By-Laws be amended by the addition of the words "Assistant Secretary" after the word "Secretary," and the addition of the words "Assistant Treasurer" after the word "Treasurer." Motion carried.

Upon motion of Dr. Abbott it was voted that this year the Treasurer and Secretary be allowed to name their own assistants.

Upon motion of Dr. Bartlett it was voted that the Society pay Dr. J. B. Samo the sum of $100 for his past services as Librarian.

Upon motion of Dr. C. A. Ring it was voted that Drs. S. G. Dorr, C. Diehl, E. T. Dorland, John Cronyn, C. G. Stockton, J. B. Andrews, F. S. Crego, H. Lapp, W. H. Gail, G. Abbott, F. F. Hoyer and F. H. Stanbro constitute a committee to take into consideration such laws and regulations as will bring about the best care and treatment of habitual drunkards.

Upon motion of Dr. Crego the following resolution was adopted:

Whereas, The Legislature of New York has enacted a law creating boards whose duties are to license properly educated physicians who have been legally graduated; and,

Whereas, The general features of this law had their inception in the Medical Society of the County of Erie that formulated a similar measure in 1883, which was introduced into the Legislature by the Medical Society of the State of New York:

*Therefore*, Be it resolved that the Medical Society of the County of Erie, in annual session assembled, desires to express its approbation of the general plan of separating the teaching and licensing authorities, as provided for in the aforementioned law.

This Society desires to congratulate the Medical Society of the State of New York, and all the friends of reform in medical education, upon the success that has attended their efforts, after these seven years of constant labor, and pledges itself to any assistance in its power to make the law effective and bring substantial results.

Upon motion of Dr. Hubbell, it was voted that the President and Secretary be authorized to appoint delegates to the Medical Association of Central New York, and issue certificates of delegation to those who may desire to act as such.

Upon motion of Dr. Crego, it was voted that the President appoint a committee of ten to act as entertainment committee and committee of arrangements, to entertain the members of the Medical Association of Central New York.

Upon motion it was voted that the thanks of the Society be tendered to the President, for the efficient manner in which he has presided over the meetings of the Society, and also to the other out-going officers.

Upon motion the meeting adjourned.

